# Multi-layered repair of high-flow CSF fistulae following endoscopic skull base surgery without nasal packing or lumbar drains: technical refinements to optimise outcome

**DOI:** 10.1007/s00701-023-05581-y

**Published:** 2023-04-19

**Authors:** Cathal John Hannan, Bharti Kewlani, Steven Browne, Mohsen Javadpour

**Affiliations:** 1 Manchester Centre for Clinical Neurosciences, Manchester, England UK; 2 National Neurosurgical Centre, Beaumont Hospital, Dublin, Ireland; 3 Royal College of Surgeons in Ireland, Dublin, Ireland; 4 School of Medicine, Trinity College Dublin, Dublin, Ireland

**Keywords:** CSF leak, Skull base, Endoscopic skull base, Pituitary adenoma, Meningioma, Chordoma

## Abstract

**Aims:**

Post-operative CSF leak remains a significant problem following endoscopic skull base surgery, particularly when there is a high-flow intra-operative CSF leak. Most skull base repair techniques are accompanied by the insertion of a lumbar drain and/or the use of nasal packing which have significant shortcomings. Our aim was to review the results of a large series of endoscopic skull base cases where a high-flow intra-operative CSF leak rate was encountered and repaired to assess if modifications in technique could reduce the post-operative CSF leak rate.

**Methods:**

A retrospective review of a prospectively maintained database of skull base cases performed by a single surgeon over a 10-year period was performed. Data regarding patient demographics, underlying pathology, skull base repair techniques and post-operative complications were analysed.

**Results:**

One hundred forty-two cases with high-flow intra-operative CSF leak were included in the study. The most common pathologies were craniopharyngiomas (55/142, 39%), pituitary adenomas (34/142, 24%) and meningiomas (24/142, 17%). The CSF leak rate was 7/36 (19%) when a non-standardised skull base repair technique was used. However, with the adoption of a standardised, multi-layer repair technique, the post-operative CSF leak rate decreased significantly (4/106, 4% vs. 7/36, 19%, *p* = 0.006). This improvement in the rate of post-operative CSF leak was achieved without nasal packing or lumbar drains.

**Conclusion:**

With iterative modifications to a multi-layered closure technique for high-flow intra-operative CSF leaks, it is possible to obtain a very low rate of post-operative CSF leak, without lumbar drains or nasal packing.

## Introduction

The most significant advance in the management of skull base pathology over the last 20 years has been the introduction of endoscopic endonasal approaches (EEA) to lesions of the ventral skull base [25]. These approaches serve as an alternative to open craniotomy and may, in selected circumstances, lead to higher rates of gross total resection, improved neurological outcomes, lower complication rates and shorter hospital stays [8, 18, 24].

However, a major initial shortcoming of EEA for skull base pathology was the associated high rate of post-operative cerebrospinal fluid (CSF) leak; in early published series, the incidence of this complication often exceeded 20% [35]. CSF leak is the most common source of morbidity following endoscopic skull base surgery, and can lead to the development of life-threatening intracranial infection and pneumocephalus [19, 32]. Patients undergoing expanded approaches, involving large arachnoid breaches and high-flow intra-operative CSF leaks, are particularly susceptible to this complication [5, 13]. The adoption of the nasoseptal flap (NSF) was instrumental in attempts to reduce the incidence of post-operative CSF leak, and it is now commonly used in skull base repair following EEA [9, 25].

Multiple skull base repair protocols have been described, and a review of these techniques and their associated results has recently been published by our group [11]. In addition to the use of a synthetic or autologous dural substitute and a NSF, these techniques are often supplemented with intra-nasal packing or lumbar drainage (LD) [7, 36, 37]. Our current practice involves the use of autologous fascia lata and a NSF, secured with BioGlue® (CryoLife Inc., USA), without the requirement for any nasal packing or adjunctive lumbar drainage [11]. We have previously described the results of our skull base repair technique in 270 patients with all grades of intra-operative CSF leak, and reported a CSF leak rate of 1% in the latter third of the series [10]. This study focuses on the results of our skull base repair protocol following EEA when a high-flow intra-operative CSF leak was encountered, and describes the adaptions in our repair protocol that have led us to the current iteration of our skull base repair protocol.

## Materials and methods

All endoscopic skull base cases performed by a single surgeon, where an intra-operative high-flow CSF leak was encountered between January 2012 and August 2022, were recorded prospectively in a clinical database and included in this study. Intra-operative CSF leaks were graded according to the Kelly grading system and cases with grade 3 leaks ‘large diaphragmatic and/or dural defect’ were included [5]. For all pathologies except pituitary adenomas, expanded approaches beyond the sella were used. Post-operative CSF leaks were diagnosed by the ‘tilt’ test in keeping with other studies in this area [3]. The incidence of post-operative meningitis and sinusitis was obtained via retrospective review of the medical records.

**Fig. 1:**
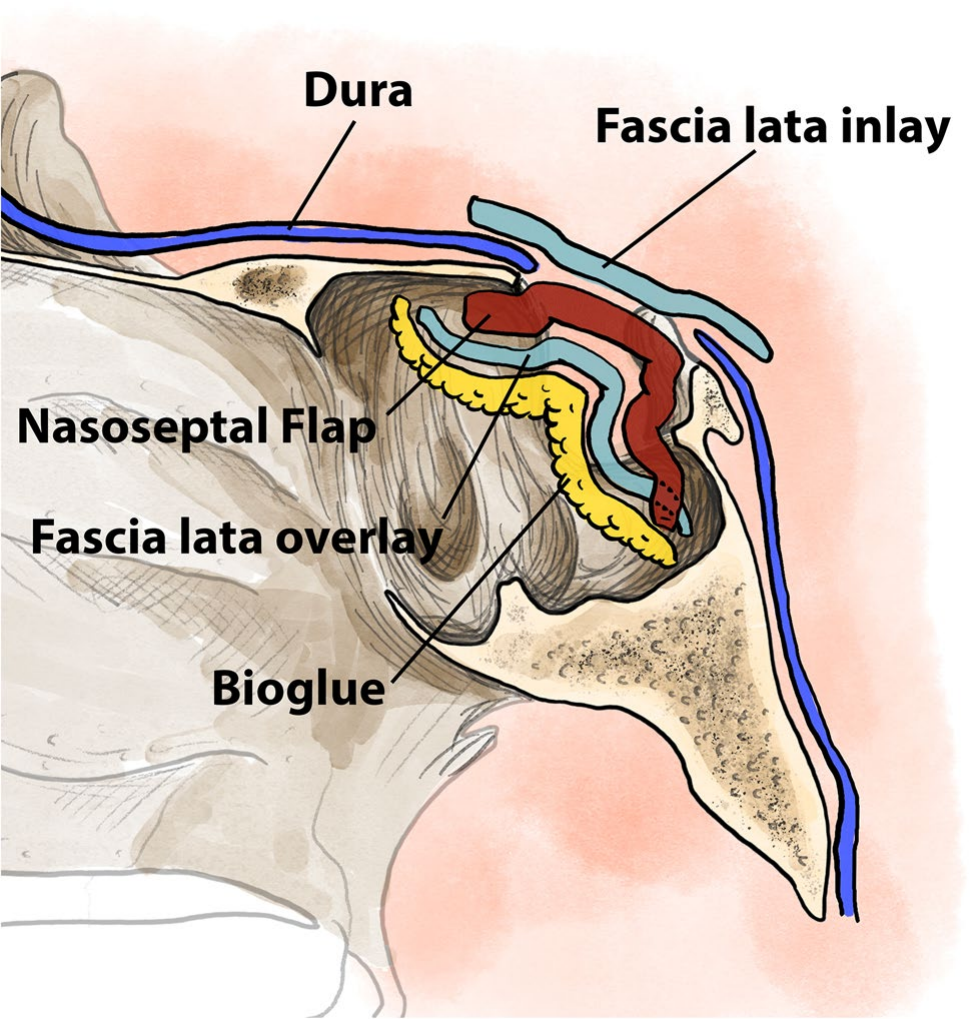
Diagrammatic representation of the materials used in skull base repair and the order in which they are placed in the standardised technique. The most recent iteration consists of an inlay fascia lata graft, covered by a nasoseptal flap which is then covered with a further layer of fascia lata and secured using BioGlue® tissue sealant

Our skull base repair technique has undergone several modifications over the 10-year period studied. Our most commonly used technique has involved the use of 2 layers of fascia lata, NSF and a dural sealant. This ‘standardised technique’ involves multi-layer closure with the placement of an inlay (intradural) layer of endogenous fascia lata, with a NSF placed over the bony and dural defects. Following this, a further layer of fascia lata is placed over the NSF, and the entire construct is secured with BioGlue® tissue sealant, applied extradurally, as described previously [11] (Fig. 1). Previously, we have also placed an onlay (extradural) fascia lata graft prior to placement of the NSF, which was then subsequently covered by the NSF and secured with BioGlue®.

The ‘non-standardised technique’, rather than being a single, unified technique, reflects a time period when non-standardised techniques were used in the earlier part of the series and involved a variety of combinations of intradural placement of fat graft, synthetic dural substitute, Dura-Guard (Baxter, USA) or Duramend (Collagen Matrix, USA) or endogenous rectus sheath which was covered by a NSF and secured with BioGlue® or DuraSeal® (Integra LifeSciences, USA).

No nasal tampons or Foley catheters were used to buttress the skull base repair, and LDs were not utilised as an adjunct to any of the repair techniques.

All statistical analysis was performed in SPSS version 25 (IBM, USA). The *χ*^2^ (with Yate’s correction where necessary) and Fisher’s exact tests were used for intergroup comparisons of categorical variables. Bonferroni corrections were used to correct for multiple comparisons. This project was approved by our institutional audit board and the requirement for individual patient consent was waived, as per institutional policy. The STROBE guidelines were followed in the drafting of this manuscript [33].

## Results

During the study period (January 2012–August 2022), 541 EEAs were performed by a single surgeon. Of these, 142 EEA resulting in a high-flow CSF were performed. Table 1 provides a breakdown of the demographic details, underlying diagnosis and post-operative complications by repair technique implemented. Craniopharyngiomas (39%), pituitary adenomas (24%) and meningiomas (17%) accounted for the majority of cases in this series, with the remainder composed of other pathologies as described in Table 1.

**Table 1:** Demographic and clinical characteristics of overall patient cohort, broken down by skull base repair technique employed. Figures in parentheses indicate interquartile range or percentages, as indicated

Characteristics	Total (*n* = 142)	‘Non-standardised technique’ (*n* = 36)	Standardised technique (*n* = 106)
Female (%)	71 (50)	20 (56)	51 (48)
Median age (IQR)	47 (37)	44 (35)	47 (40)
Median BMI (IQR)	28 (11)	33 (15)	28 (10)
Re-operation (%)	32 (23)	14 (40)	17 (16)
Previous radiotherapy (%)	14 (10)	7 (19)	7 (6)
Pituitary adenoma (%)	34 (24)	12 (33)	22 (21)
Craniopharyngioma (%)	55 (39)	13 (36)	42 (40)
Meningioma (%)	24 (17)	2 (5)	22 (21)
Chordoma/chondrosarcoma (%)	10 (7)	1 (3)	9 (8)
Rathke’s cleft cyst (%)	4 (3)	2 (5)	2 (2)
Other (%)	15 (11)	6 (17)	9 (8)
Transplanar/transtubercular approach (%)	84 (59)	17 (47)	67 (63)
Transellar approach (%)	48 (33)	18 (50)	30 (28)
Transclival approach (%)	10 (7)	1 (3)	9 (8)
Post-op CSF leak (%)	11 (8)	7 (19)	4 (4)
Post-op meningitis (%)	5 (4)	3 (8)	2 (2)

**Fig. 2:**
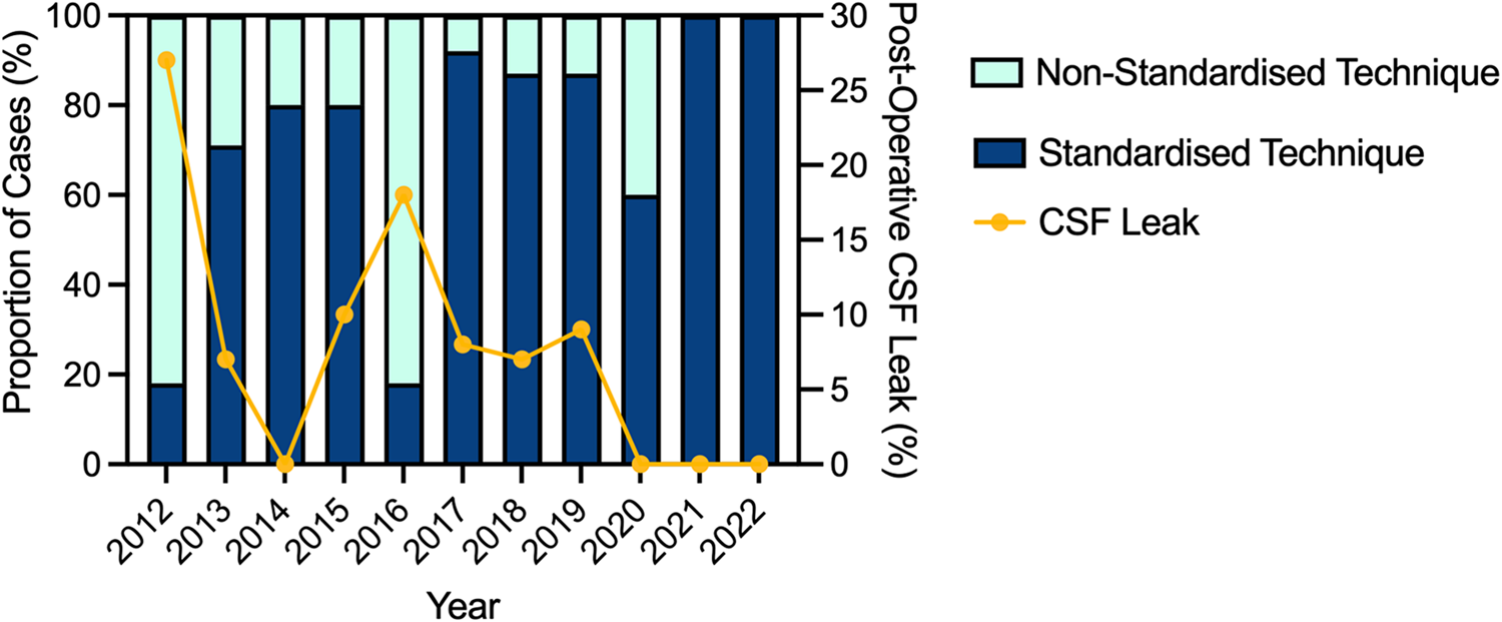
Stacked column graph indicating the proportion of cases per year repaired using the standardised and non-standardised repair techniques (left *y* axis). The line graph overlain on the columns indicates the proportion of cases where a post-operative CSF leak was encountered (right *y* axis). There was an increase in the use of standardised techniques as the series progressed and a corresponding decrease in the rates of post-operative CSF leak

The rate of post-operative CSF leak with our standardised technique was 4/104 (4%). This was significantly lower than the post-operative CSF leak rate associated with the non-standardised technique which was 7/36 (19%). Table 2 demonstrates the results of univariate analyses of potential predictors of post-operative CSF leak. Use of a standardised repair technique, rather than the non-standardised technique, was the only factor associated with a significant decrease in the rate of post-operative CSF leak (4/106, 4% vs. 7/36, 19% Fisher’s exact test, *p* = 0.006). Surgical approach used (i.e. transtubercular, transsellar, transclival) had no impact on the rate of post-operative CSF leak. The standardised technique was used significantly more commonly as the series progressed (*χ*^2^ = 52.59, *p* ≤ 0.001), and the non-standardised technique was last used in 2020. Figure 2 illustrates these trends in the data.

**Table 2:** Results of univariate analysis of factors potentially associated with post-operative CSF leak. Statistically significant comparisons indicated by bold font

Factors	Rate of post-operative CSF leak	Univariate analysis
Age		
≥ 50	3/61 (5%)	Fisher’s exact test, *p* = 0.274
< 50	8/81 (10%)	
Gender		
Female	4/71 (6%)	*χ*^2^ = 0.887, *p* = 0.346
Male	7/71 (10%)	
Obesity*		
Obese (BMI ≥ 30 kg/m^2^)	5/49 (10%)	Fisher’s exact test, *p* = 0.487
Non-obese (BMI < 30 kg/m^2^)	4/69 (6%)	
Year of surgery		
2012–2014	4/40 (10%)	Fisher’s exact test, *p* = 0.344
2015–2018	5/47 (11%)	
2019–2022	2/55 (4%)	
Previous surgery		
Yes	3/32 (10%)	Fisher’s exact test, *p* = 0.701
No	8/110 (7%)	
Previous radiotherapy		
Yes	2/15 (14%)	Fisher’s exact test, *p* = 0.299
No	9/127 (7%)	
Pathology		
Pituitary adenoma	1/34 (3%)	Fisher’s exact test, *p* = 0.198
Craniopharyngioma	6/55 (11%)	
Meningioma	1/24 (4%)	
Chordoma	2/10 (20%)	
Rathke’s cleft cyst	1/4 (25%)	
Other	0/15 (0%)	
Surgical approach		
Transplanar/transtubercular	7/84 (8%)	Fisher’s exact test, *p* = 0.179
Transellar	2/48 (2%)	
Transclival	2/10 (20%)	
Repair technique		
Non-standardised	7/36 (19%)	**Fisher’s exact test, *p* = 0.006**
Standardised	4/106 (4%)	

^*^BMI data available for 118 patients

**Fig. 3:**
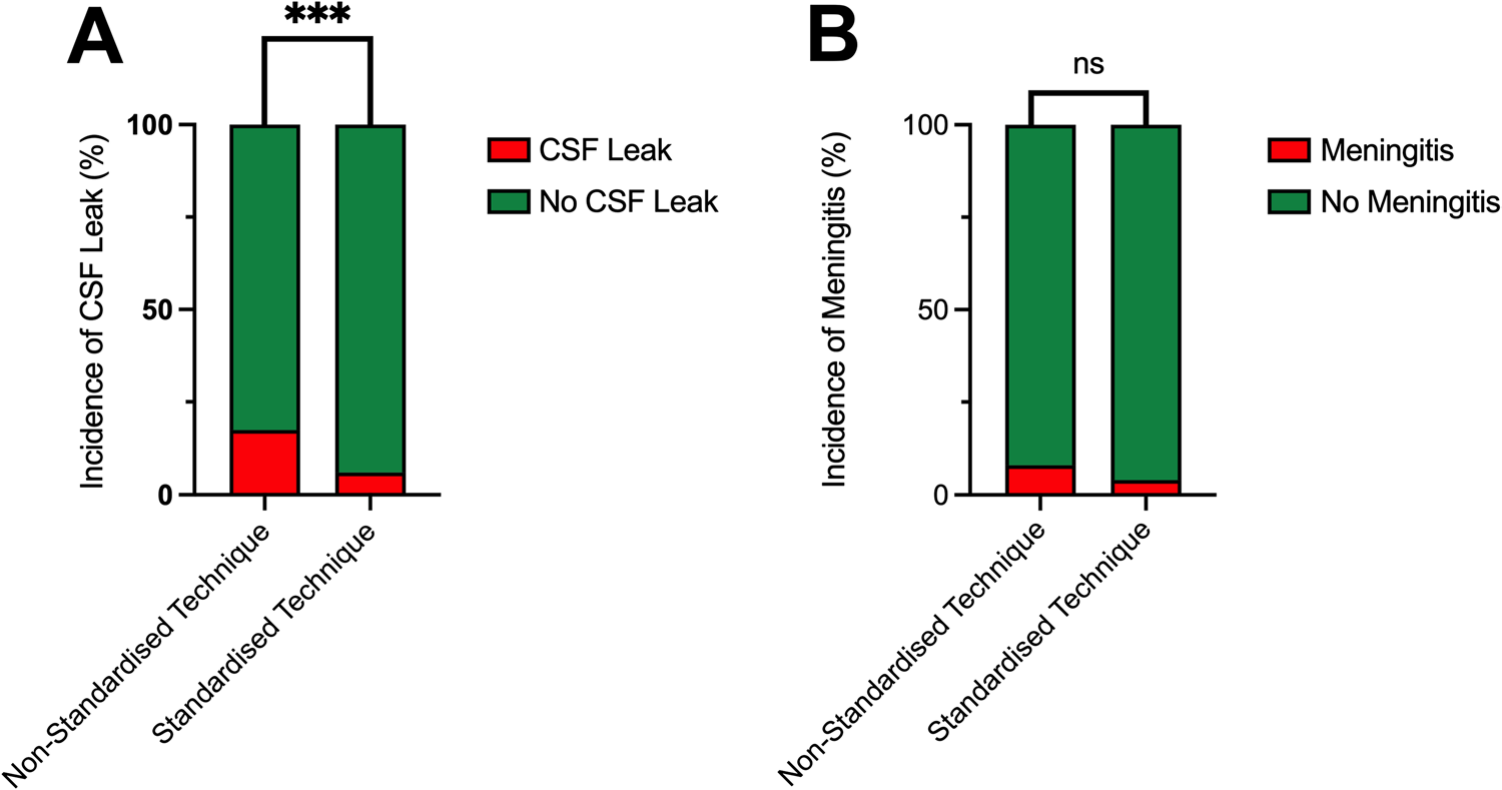
Stacked column graphs indicating the proportion of cases complicated by (**A**) post-operative CSF leak or (**B**) post-operative meningitis. ***p ≤ 0.001; ns, no statistically significant difference

There was no significant difference in the incidence of post-operative meningitis when all three techniques were compared (technique 1, 3/36, 8%; technique 2, 2/66, 3%; technique 3, 0/40, 0%; Fisher’s exact Test, *p* = 0.372) (Fig. 3). There were no cases of post-operative visual deterioration in this cohort.

## Discussion

The results of this study demonstrate that with appropriate adaptions in skull base repair protocol, it is possible to significantly reduce the post-operative CSF leak rate following EEA, even in the presence of high-flow intra-operative CSF leaks. These results were achieved by means of iterative modifications to an existing repair protocol utilising multi-layered closure including a NSF and without the requirement for nasal packing or prophylactic LD insertion. The post-operative CSF leak rate in the early part of our series using a non-standardised approach was 19%. After introduction of standardised techniques, the post-operative CSF leak rate dropped to 4%. On univariate analysis, the only factor found to be predictive of post-operative CSF leak in this series was the repair technique used: the use of standardised repair techniques was associated with a significant decrease in the rate of post-operative CSF leak when compared to a non-standardised repair technique.

**Fig. 4:**
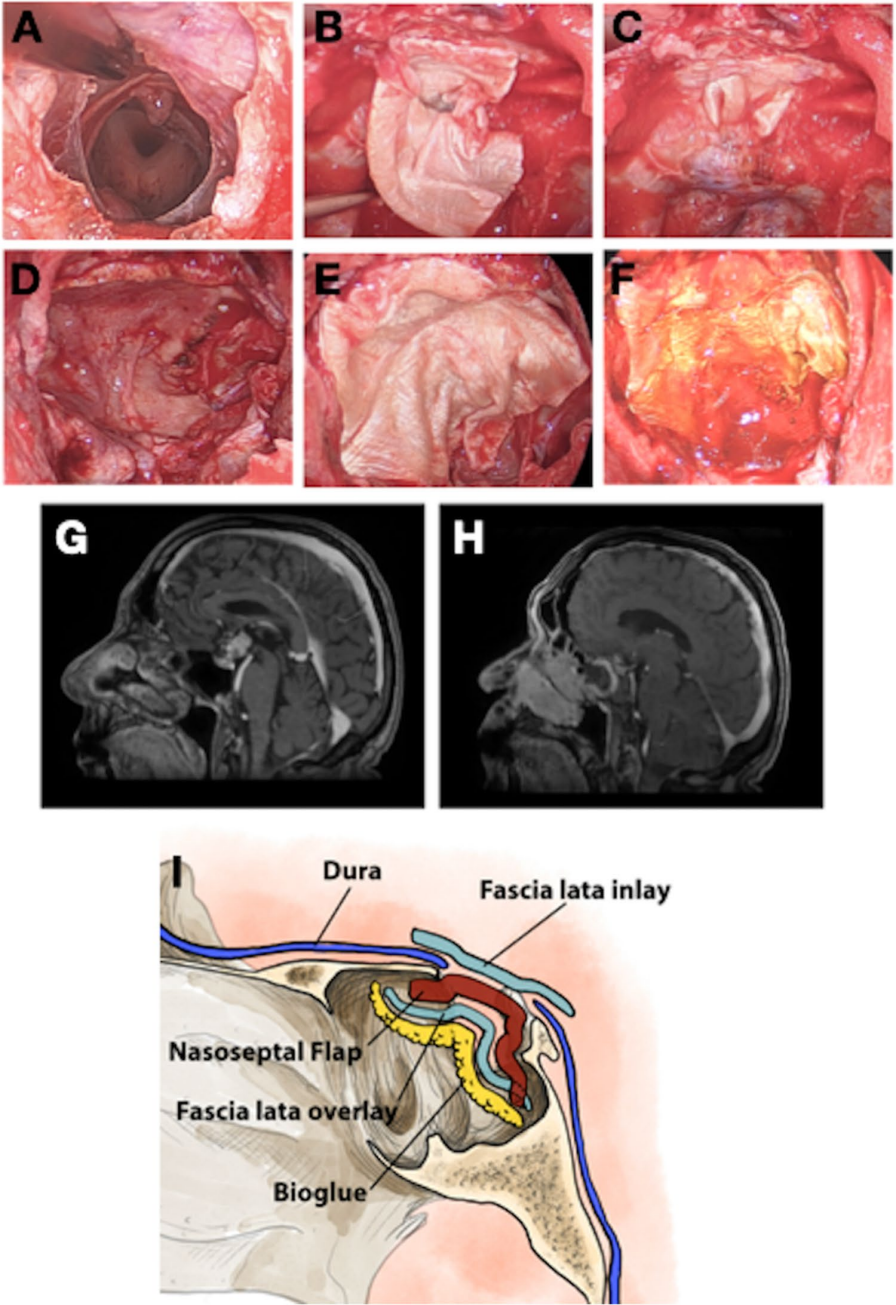
Multi-panelled figure demonstrating the repair of a defect in the tuberculum sella/planum sphenoidale following the resection of a suprasellar craniopharyngioma. **A** Intra-operative endoscopic photograph demonstrating the skull base defect and view into the third ventricle following tumour resection. **B** Endoscopic photograph demonstrating the placement of an inlay fascia lata graft. Note that this graft is significantly larger than the dural defect. **C** Endoscopic photograph demonstrating the final position of the fascia lata graft, with the edges tucked in underneath the margins of the skull base defect. **D** Endoscopic photograph demonstrating placement of a pedicled nasoseptal flap to completely cover the skull base defect followed by the (**E**) placement of an onlay fascia lata graft which is in turn secured with (**F**) a layer of BioGlue. **G** Pre-operative T1-weighted MRI scan demonstrating the presence of a suprasellar craniopharyngioma with superior extension into the third ventricle. **H** Post-operative T1-weighted MRI scan demonstrating complete tumour resection and the enhancement of the pedicled nasoseptal flap overlying the skull base defect. **I** Diagrammatic representation of the Dublin technique as applied to approaches to sellar/suprasellar pathology

**Fig. 5:**
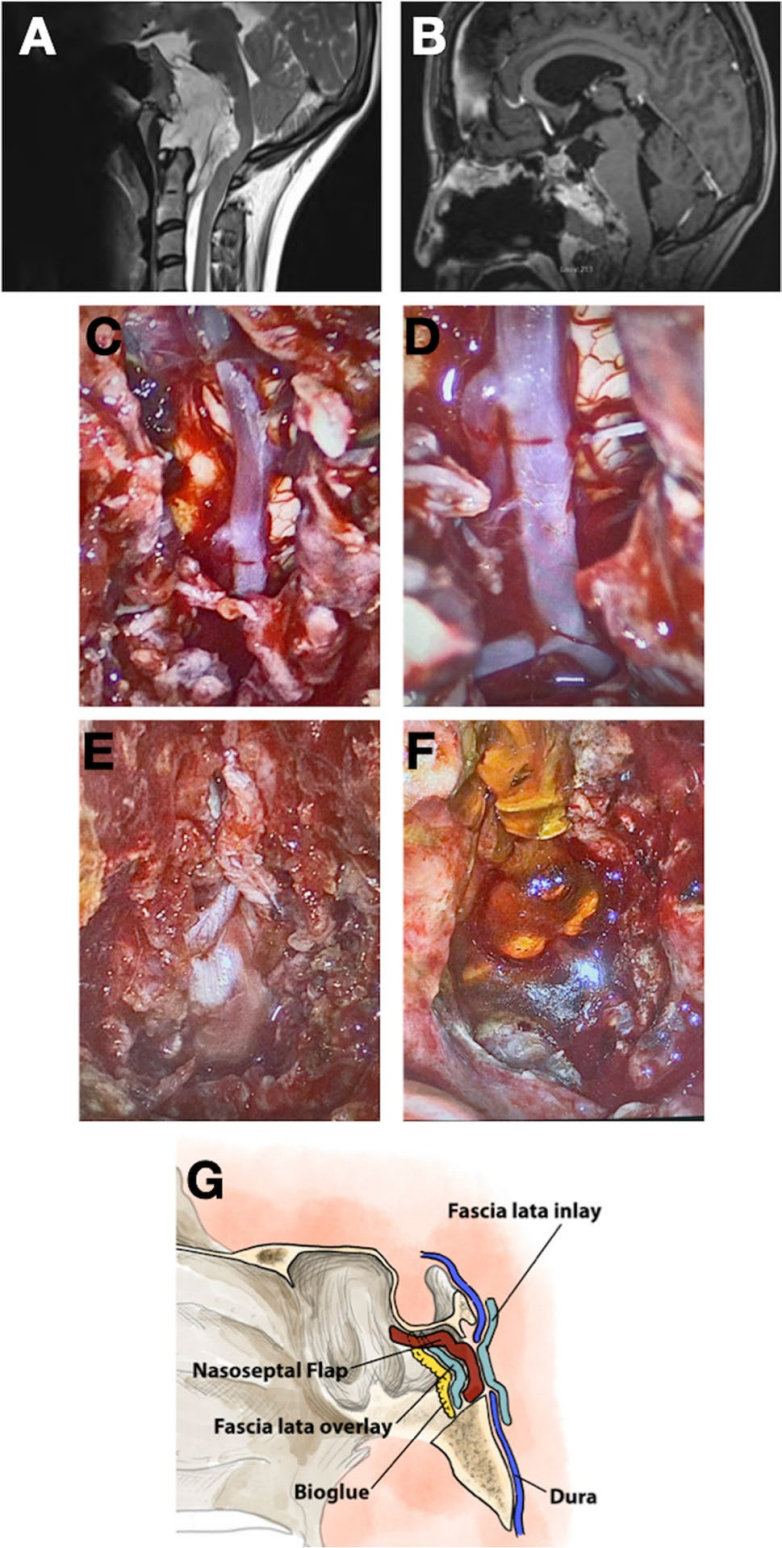
Multi-panelled figure demonstrating the repair of a clival defect following the resection of a chordoma. **A** Pre-operative T2-weighted MRI scan demonstrating the presence of an extensive clival chordoma, causing severe brainstem compression. **B** Post-operative T1-weighted MRI scan following contrast administration, demonstrating complete tumour resection with resolution of brainstem compression, along with enhancement of the nasoseptal flap. **C** Intra-operative endoscopic photograph following tumour resection, demonstrating a longitudinally extensive clival defect. The basilar artery and brainstem are clearly displayed. **D** Endoscopic view of the vertebro-basilar junction and emergence of the right anterior inferior cerebellar artery. **E** Intra-operative photograph demonstrating placement of the inlay layer of fascia lata within the dural/bony defect. **F** Final overview of the skull base repair, with a layer of BioGlue applied over an onlay fascia lata graft. **G** Diagrammatic representation of the Dublin technique as applied to approaches to clival pathology

Undoubtedly, the introduction of the pedicled NSF represented a major technical advance in the field of endoscopic skull base surgery, and facilitated the continued evolution of the indications for EEA to include extra-sellar skull base pathology, which may not otherwise have been possible due to the prohibitively high rates of CSF leak [9, 20]. The widespread adoption of the NSF coincided with a reduction in the CSF leak rate from ~ 20 to ~ 5% in the published literature, and further developments since then have been largely iterative, with CSF leak rates following EEA with all degrees of intra-operative CSF leak rate varying between 2 and 4% [2, 3, 7, 14, 16, 21, 22, 31, 36]. The NSF has now been incorporated into a number of skull base repair techniques, often accompanied by adjuncts intended to provide additional support to the repair. Garcia-Navarro et al. previously described one such technique known as the ‘Gasket Seal Closure’ that resulted in a post-operative CSF leak rate of 4.5% [7]. In their publication, the authors argued that the placement of the solid buttress was essential, and that this avoided the need for nasal packing which is uncomfortable for the patient. Similarly, Conger et al. reported the results of skull base repair in 551 cases, and argued that a solid buttress was required to bolster the repair in the setting of a high-flow intra-operative CSF leak. In our series, we have demonstrated that the use of this solid buttress may not be necessary, even in the presence of large skull base defects with significant dural defects (Figs. 4 and 5).

Our use of BioGlue™ (CryoLife, USA) tissue sealant, which has a demonstrably higher tensile strength than other tissue sealants, may potentially be the factor that obviated the need for a buttress to reinforce the skull base repair [26]. However, we did not compare this with other tissue sealants and therefore no definite conclusions can be made regarding the most optimal type of tissue sealant. It must also be noted that previous concerns have been raised in the literature regarding an increased rate of CSF fistulae and post-operative infection in association with the use of BioGlue®. We have previously published a study that demonstrated no increase in the rate of post-operative infection or sino-nasal morbidity when this product was used following endoscopic transsphenoidal surgery [6, 12, 30].

Prophylactic LDs are used by many skull base surgeons in the post-operative period with the aim of reducing the risk of post-operative CSF leaks [11]. Even surgeons who do not use LDs routinely in endoscopic skull base surgery tend to use it in all or at least in selected patients with high-flow intra-operative leaks; a recently published multi-centre analysis of skull base repair protocols following EEA identified that prophylactic LDs were used in 41% of cases where a high-flow intra-operative CSF leak was encountered [4, 7, 28]. A randomised controlled trial (RCT) found the use of LDs to be beneficial in endoscopic skull base surgery with high-flow intra-operative leaks, reducing the risk of post-operative CSF leak from 21 to 8% [37]. However, LDs have also been associated with significant morbidity including low-pressure headache, nausea and vomiting in 13–63%; meningitis and other infections in 4–10%; radiculopathy; overdrainage with risk of subdural haemorrhage; and complications associated with decreased patient mobilisation [1, 27, 29]. Interestingly, a further RCT of LD vs. no LD following endoscopic skull base surgery with a high-flow intra-operative CSF leak demonstrated no difference in the rates of post-operative CSF leak, but a markedly increased rate of complications and length of stay for those patients assigned to the LD group [15]. Using our standardised technique, we have achieved a low post-operative CSF leak rate in patients with high-flow intra-operative CSF leaks, without the use of LDs in any of our patients. The lower post-operative leak rates in our series compared to the rates obtained in the RCT by Zwagerman et al. may be due to differences in repair technique [37]. However, the proportion of patients with posterior fossa pathology (such as chordoma and chondrosarcoma) was much lower in our series (7% vs 29%), which may act as a confounder given the technical difficulty often encountered in achieving watertight closure of extensive clival defects.

From our perspective, we feel that the placement of an inlay fascia lata graft as a dural substitute, beyond the margins of the bony/dural defect, is of critical importance. A fascia lata graft larger than the dural defect is placed intradurally, and opposed to the internal surface of the skull base. In our view, the placement of this inlay graft allows the natural brain/CSF pulsations to push the graft against the dura mater with each pulsation, as opposed to the risk of an extradural onlay graft migrating away from the defect. We have not observed any migration of the fascia lata away from the dural defect, which was the rationale for the creation of the ‘Bilayer Button’ by Luginbuhl et al. [22]. While the benefits of the NSF in the prevention of a post-operative CSF leak have been well documented previously, a nuance of the skull base repair technique we feel is particularly important is the direct apposition of endogenous tissues to each other, as in our view they adhere to each other much better than endogenous tissues to do synthetic materials such as dural substitutes [2, 3, 7, 14, 16, 21, 22, 31, 36]. Furthermore, although we cannot provide any empirical evidence in support of this assertion, we are of the view that one should not permit synthetic repair materials such as tissue sealant or haemostatic agents to intercede between the fascia lata and NSF, as these may act to prevent the adherence of these endogenous graft materials to each other. As mentioned previously, BioGlue® forms an extremely firm supportive layer once set, which counteracts the CSF pulsations which may otherwise lead to failure of the repair. Moreover, our repair technique does not involve the placement of any nasal packing or catheters. It has been observed that patients find the placement and removal of nasal packing material to be the most uncomfortable part of the recovery from EEA to skull base lesions, and we agree with the developers of the ‘Gasket Seal Closure’ that the optimal skull base repair protocol should avoid this [23]. Finally, although there is some evidence to suggest that prophylactic lumbar drains are efficacious in the prevention of post-operative CSF leak, due to the potential for significant complications, we believe that their use is best avoided if possible [1, 27, 29, 37].

The strengths of this study include its prospective nature, and that consecutive cases were included, limiting the influence of any selection bias. The study is further strengthened by the use of robust, validated technique for the grading of intra-operative CSF leaks [5]. The wide variety of pathology included (Table 1) serves to broaden the applicability of the results, and make them applicable to any EEA where a high-flow CSF leak is encountered. A potential confounding factor in this study was that increasing surgical experience, rather than any improvement in surgical technique, may have been responsible for the decrease in post-operative CSF leak. We specifically assessed for a potential influence of increasing experience on univariate analysis (Table 2), but the only factor significantly associated with the incidence of this complication was the skull base repair technique. Moreover, the senior author had been practicing as an independent consultant neurosurgeon for 5 years at the time of the initiation of this series, and may have already overcome the learning curve associated with EEA [17]. Although it has been argued that continued improvement in outcome can be observed at the ‘tail end’ of the learning curve, the publication introducing this concept found no impact of further experience beyond 200 cases on the rate of post-operative CSF leak rates, and this finding has recently been replicated in a further study of over 1000 EEA procedures [13, 34]. Undoubtedly, this study is limited by the fact that the results represent those of a single-surgeon, single-institution series, and these results may not be replicable in other settings. We intend to validate the results of the present study with a multi-centre, multi-surgeon study in the future.

## Conclusions

With iterative modifications to a multi-layered closure technique based on the use of a vascularised NSF, it is possible to obtain very low rates of post-operative CSF leak, even when a high-flow intra-operative CSF leak is encountered. This was achieved without the use of LDs and/or nasal packing, suggesting that these invasive adjuncts may not be necessary.

## Data Availability

Data is available from the authors on request.

## Code availability

N/A.

## References

[1] Ackerman PD, Spencer DA, Prabhu VC (2013). The efficacy and safety of preoperative lumbar drain placement in anterior skull base surgery. J Neurol Surg Rep.

[2] Cavallo LM, Solari D, Somma T, Cappabianca P (2019). The 3F (fat, flap, and flash) technique for skull base reconstruction after endoscopic endonasal suprasellar approach. World Neurosurg.

[3] Conger A, Zhao F, Wang X, Eisenberg A, Griffiths C, Esposito F, Carrau RL, Barkhoudarian G, Kelly DF (2018). Evolution of the graded repair of CSF leaks and skull base defects in endonasal endoscopic tumor surgery: trends in repair failure and meningitis rates in 509 patients. J Neurosurg.

[4] CSF rhinorrhoea after endonasal intervention to the skull base (CRANIAL): a multicentre prospective observational study (2022). Front Oncol 12:1049627. 10.3389/fonc.2022.1049627 10.3389/fonc.2022.1049627 PMC9846732 36688936

[5] Esposito F, Dusick JR, Fatemi N, Kelly DF (2007). Graded repair of cranial base defects and cerebrospinal fluid leaks in transsphenoidal surgery. Operative Neurosurgery.

[6] Gaberel T, Borgey F, Thibon P, Lesteven C, Lecoutour X, Emery E (2011). Surgical site infection associated with the use of bovine serum albumine-glutaraldehyde surgical adhesive (BioGlue) in cranial surgery: a case-control study. Acta Neurochir (Wien).

[7] Garcia-Navarro V, Anand VK, Schwartz TH (2013). Gasket seal closure for extended endonasal endoscopic skull base surgery: efficacy in a large case series. World Neurosurg.

[8] Graffeo CS, Dietrich AR, Grobelny B, Zhang M, Goldberg JD, Golfinos JG, Lebowitz R, Kleinberg D, Placantonakis DG (2014). A panoramic view of the skull base: systematic review of open and endoscopic endonasal approaches to four tumors. Pituitary.

[9] Hadad G, Bassagasteguy L, Carrau RL, Mataza JC, Kassam A, Snyderman CH, Mintz A (2006). A novel reconstructive technique after endoscopic expanded endonasal approaches: vascular pedicle nasoseptal flap. Laryngoscope.

[10] Hannan CJ, Almhanedi H, Al-Mahfoudh R, Bhojak M, Looby S, Javadpour M (2020). Predicting post-operative cerebrospinal fluid (CSF) leak following endoscopic transnasal pituitary and anterior skull base surgery: a multivariate analysis. Acta Neurochir (Wien).

[11] Hannan CJ, Kelleher E, Javadpour M (2020). Methods of skull base repair following endoscopic endonasal tumor resection: a review. Front Oncol.

[12] Hannan CJ, Nolan D, Corr P, Amoo M, Murray D, Looby S, Javadpour M (2022). Sinonasal outcomes associated with the use of BioGlue® in endoscopic transsphenoidal pituitary surgery. Neurosurg Rev.

[13] Hardesty DA, Montaser A, Kreatsoulas D, Shah VS, VanKoevering KK, Otto BA, Carrau RL, Prevedello DM (2021) Complications after 1002 endoscopic endonasal approach procedures at a single center: lessons learned, 2010–2018. J Neurosurg 1–12. 10.3171/2020.11.Jns202494 10.3171/2020.11.JNS202494 PMC10193480 34359021

[14] Horiguchi K, Murai H, Hasegawa Y, Hanazawa T, Yamakami I, Saeki N (2010). Endoscopic endonasal skull base reconstruction using a nasal septal flap: surgical results and comparison with previous reconstructions. Neurosurg Rev.

[15] Huo CW, King J, Goldschlager T, Dixon B, Chen Zhao Y, Uren B, Wang YY (2022). The effects of cerebrospinal fluid (CSF) diversion on post-operative CSF leak following extended endoscopic anterior skull base surgery. J Clin Neurosci.

[16] Kassam AB, Thomas A, Carrau RL, Snyderman CH, Vescan A, Prevedello D, Mintz A, Gardner P (2008). Endoscopic reconstruction of the cranial base using a pedicled nasoseptal flap. Neurosurgery.

[17] Koc K, Anik I, Ozdamar D, Cabuk B, Keskin G, Ceylan S (2006). The learning curve in endoscopic pituitary surgery and our experience. Neurosurg Rev.

[18] Labidi M, Watanabe K, Bouazza S, Bresson D, Bernat AL, George B, Froelich S (2016). Clivus chordomas: a systematic review and meta-analysis of contemporary surgical management. J Neurosurg Sci.

[19] Lai LT, Trooboff S, Morgan MK, Harvey RJ (2014). The risk of meningitis following expanded endoscopic endonasal skull base surgery: a systematic review. J Neurol Surg B Skull Base.

[20] Laws ER, Kanter AS, Jane JA, Dumont AS (2005). Extended transsphenoidal approach. J Neurosurg.

[21] Liu JK, Schmidt RF, Choudhry OJ, Shukla PA, Eloy JA (2012). Surgical nuances for nasoseptal flap reconstruction of cranial base defects with high-flow cerebrospinal fluid leaks after endoscopic skull base surgery. Neurosurg Focus.

[22] Luginbuhl AJ, Campbell PG, Evans J, Rosen M (2010). Endoscopic repair of high-flow cranial base defects using a bilayer button. Laryngoscope.

[23] Lwu S, Edem I, Banton B, Bernstein M, Vescan A, Gentili F, Zadeh G (2012). Quality of life after transsphenoidal pituitary surgery: a qualitative study. Acta Neurochir (Wien).

[24] Magill ST, Morshed RA, Lucas CG, Aghi MK, Theodosopoulos PV, Berger MS, de Divitiis O, Solari D, Cappabianca P, Cavallo LM, McDermott MW (2018). Tuberculum sellae meningiomas: grading scale to assess surgical outcomes using the transcranial versus transsphenoidal approach. Neurosurg Focus.

[25] Martinez-Perez R, Requena LC, Carrau RL, Prevedello DM (2021). Modern endoscopic skull base neurosurgery. J Neurooncol.

[26] Murdock MH, Chang JT, Luketich SK, Pedersen D, Hussey GS, D'Amore A, Badylak SF (2019). Cytocompatibility and mechanical properties of surgical sealants for cardiovascular applications. J Thorac Cardiovasc Surg.

[27] Ransom ER, Palmer JN, Kennedy DW, Chiu AG (2011). Assessing risk/benefit of lumbar drain use for endoscopic skull-base surgery. Int Forum Allergy Rhinol.

[28] Roxbury CR, Lobo BC, Kshettry VR, D'Anza B, Woodard TD, Recinos PF, Snyderman CH, Sindwani R (2018). Perioperative management in endoscopic endonasal skull-base surgery: a survey of the North American Skull Base Society. Int Forum Allergy Rhinol.

[29] Scheithauer S, Bürgel U, Bickenbach J, Häfner H, Haase G, Waitschies B, Reinges MH, Lemmen SW (2010). External ventricular and lumbar drainage-associated meningoventriculitis: prospective analysis of time-dependent infection rates and risk factor analysis. Infection.

[30] Sen A, Green KM, Khan MI, Saeed SR, Ramsden RT (2006). Cerebrospinal fluid leak rate after the use of BioGlue in translabyrinthine vestibular schwannoma surgery: a prospective study. Otol Neurotol.

[31] Thorp BD, Sreenath SB, Ebert CS, Zanation AM (2014). Endoscopic skull base reconstruction: a review and clinical case series of 152 vascularized flaps used for surgical skull base defects in the setting of intraoperative cerebrospinal fluid leak. Neurosurg Focus.

[32] Vengerovich G, Park KW, Antoury L, Wells C, Suh JD, Lee JT, Heaney AP, Bergsneider M, Wang MB (2020). Readmissions after endoscopic skull base surgery: associated risk factors and prevention. Int Forum Allergy Rhinol.

[33] von Elm E, Altman DG, Egger M, Pocock SJ, Gøtzsche PC, Vandenbroucke JP (2007). The Strengthening the Reporting of Observational Studies in Epidemiology (STROBE) statement: guidelines for reporting observational studies. Ann Intern Med.

[34] Younus I, Gerges MM, Uribe-Cardenas R, Morgenstern PF, Eljalby M, Tabaee A, Greenfield JP, Kacker A, Anand VK, Schwartz TH (2020). How long is the tail end of the learning curve? Results from 1000 consecutive endoscopic endonasal skull base cases following the initial 200 cases. J Neurosurg.

[35] ZamanipoorNajafabadi AH, Khan DZ, Muskens IS, Broekman MLD, Dorward NL, van Furth WR, Marcus HJ (2021). Trends in cerebrospinal fluid leak rates following the extended endoscopic endonasal approach for anterior skull base meningioma: a meta-analysis over the last 20 years. Acta Neurochir (Wien).

[36] Zanation AM, Carrau RL, Snyderman CH, Germanwala AV, Gardner PA, Prevedello DM, Kassam AB (2009). Nasoseptal flap reconstruction of high flow intraoperative cerebral spinal fluid leaks during endoscopic skull base surgery. Am J Rhinol Allergy.

[37] Zwagerman NT, Wang EW, Shin SS, Chang YF, Fernandez-Miranda JC, Snyderman CH, Gardner PA (2018) Does lumbar drainage reduce postoperative cerebrospinal fluid leak after endoscopic endonasal skull base surgery? A prospective, randomized controlled trial. J Neurosurg 1–7. 10.3171/2018.4.Jns172447 10.3171/2018.4.JNS172447 30485224

